# UBE2C promotes leptomeningeal dissemination and is a therapeutic target in brain metastatic disease

**DOI:** 10.1093/noajnl/vdad048

**Published:** 2023-04-28

**Authors:** Eunice Paisana, Rita Cascão, Carlos Custódia, Nan Qin, Daniel Picard, David Pauck, Tânia Carvalho, Pedro Ruivo, Clara Barreto, Delfim Doutel, José Cabeçadas, Rafael Roque, José Pimentel, José Miguéns, Marc Remke, João T Barata, Claudia C Faria

**Affiliations:** Instituto de Medicina Molecular João Lobo Antunes, Faculdade de Medicina da Universidade de Lisboa; Av. Prof. Egas Moniz, 1649-028, Lisboa, Portugal; Instituto de Medicina Molecular João Lobo Antunes, Faculdade de Medicina da Universidade de Lisboa; Av. Prof. Egas Moniz, 1649-028, Lisboa, Portugal; Instituto de Medicina Molecular João Lobo Antunes, Faculdade de Medicina da Universidade de Lisboa; Av. Prof. Egas Moniz, 1649-028, Lisboa, Portugal; Department of Pediatric Oncology, Hematology and Clinical Immunology, Heinrich Heine University Düsseldorf, Medical Faculty, and University Hospital Düsseldorf; Moorenstraße 5, 40225 Düsseldorf, Germany; German Cancer Consortium (DKTK), Partner Site Essen/Düsseldorf, Düsseldorf, Germany; Moorenstraße 5, 40225 Düsseldorf, Germany; Department of Pediatric Oncology, Hematology and Clinical Immunology, Heinrich Heine University Düsseldorf, Medical Faculty, and University Hospital Düsseldorf; Moorenstraße 5, 40225 Düsseldorf, Germany; German Cancer Consortium (DKTK), Partner Site Essen/Düsseldorf, Düsseldorf, Germany; Moorenstraße 5, 40225 Düsseldorf, Germany; Department of Pediatric Oncology, Hematology and Clinical Immunology, Heinrich Heine University Düsseldorf, Medical Faculty, and University Hospital Düsseldorf; Moorenstraße 5, 40225 Düsseldorf, Germany; German Cancer Consortium (DKTK), Partner Site Essen/Düsseldorf, Düsseldorf, Germany; Moorenstraße 5, 40225 Düsseldorf, Germany; Instituto de Medicina Molecular João Lobo Antunes, Faculdade de Medicina da Universidade de Lisboa; Av. Prof. Egas Moniz, 1649-028, Lisboa, Portugal; Instituto de Medicina Molecular João Lobo Antunes, Faculdade de Medicina da Universidade de Lisboa; Av. Prof. Egas Moniz, 1649-028, Lisboa, Portugal; Instituto de Medicina Molecular João Lobo Antunes, Faculdade de Medicina da Universidade de Lisboa; Av. Prof. Egas Moniz, 1649-028, Lisboa, Portugal; Anatomic Pathology Department, Instituto Português de Oncologia Francisco Gentil, R. Prof. Lima Basto, 1099-023, Lisboa, Portugal; Anatomic Pathology Department, Instituto Português de Oncologia Francisco Gentil, R. Prof. Lima Basto, 1099-023, Lisboa, Portugal; Neurology Department, Laboratory of Neuropathology, Hospital de Santa Maria, Centro Hospitalar Universitário Lisboa Norte (CHULN), Av. Prof. Egas Moniz, 1649-028, Lisboa, Portugal; Neurology Department, Laboratory of Neuropathology, Hospital de Santa Maria, Centro Hospitalar Universitário Lisboa Norte (CHULN), Av. Prof. Egas Moniz, 1649-028, Lisboa, Portugal; Department of Neurosurgery, Hospital de Santa Maria, Centro Hospitalar Universitário Lisboa Norte (CHULN), Av. Prof. Egas Moniz, 1649-028, Lisboa, Portugal; Department of Pediatric Oncology, Hematology and Clinical Immunology, Heinrich Heine University Düsseldorf, Medical Faculty, and University Hospital Düsseldorf; Moorenstraße 5, 40225 Düsseldorf, Germany; German Cancer Consortium (DKTK), Partner Site Essen/Düsseldorf, Düsseldorf, Germany; Moorenstraße 5, 40225 Düsseldorf, Germany; Instituto de Medicina Molecular João Lobo Antunes, Faculdade de Medicina da Universidade de Lisboa; Av. Prof. Egas Moniz, 1649-028, Lisboa, Portugal; Instituto de Medicina Molecular João Lobo Antunes, Faculdade de Medicina da Universidade de Lisboa; Av. Prof. Egas Moniz, 1649-028, Lisboa, Portugal; Department of Neurosurgery, Hospital de Santa Maria, Centro Hospitalar Universitário Lisboa Norte (CHULN), Av. Prof. Egas Moniz, 1649-028, Lisboa, Portugal

**Keywords:** brain metastases, leptomeningeal dissemination, *UBE2C*, PI3K/mTOR inhibition

## Abstract

**Background:**

Despite current improvements in systemic cancer treatment, brain metastases (BM) remain incurable, and there is an unmet clinical need for effective targeted therapies.

**Methods:**

Here, we sought common molecular events in brain metastatic disease. RNA sequencing of thirty human BM identified the upregulation of *UBE2C*, a gene that ensures the correct transition from metaphase to anaphase, across different primary tumor origins.

**Results:**

Tissue microarray analysis of an independent BM patient cohort revealed that high expression of UBE2C was associated with decreased survival. UBE2C-driven orthotopic mouse models developed extensive leptomeningeal dissemination, likely due to increased migration and invasion. Early cancer treatment with dactolisib (dual PI3K/mTOR inhibitor) prevented the development of UBE2C-induced leptomeningeal metastases.

**Conclusions:**

Our findings reveal UBE2C as a key player in the development of metastatic brain disease and highlight PI3K/mTOR inhibition as a promising anticancer therapy to prevent late-stage metastatic brain cancer.

## Graphical Abstract

**Graphical Abstract:**
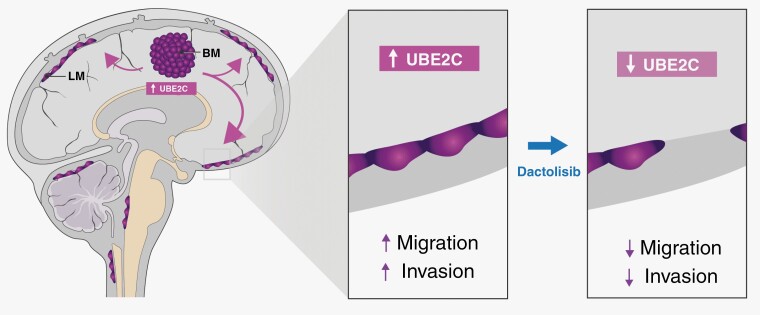

Key Points

- High levels of UBE2C in BM correlate with patients’ worse outcome.
- UBE2C promotes leptomeningeal dissemination in vivo.
- PI3K/mTOR inhibition prevents this late-stage complication of cancer.

**Importance of the Study**

Brain metastases (BM) are a late-stage complication in cancer patients and remain an incurable disease. Using RNA sequencing and tissue microarrays in surgically resected BM from cancer patients with diverse primary cancers, we have identified UBE2C as a molecular marker of prognosis in patients with brain metastatic disease. High expression of UBE2C was associated with patients’ worse survival. We have shown that UBE2C increases migration and invasion and induces leptomeningeal dissemination in vivo. Importantly, early treatment of UBE2C-driven mouse and patient-derived xenografts with the dual PI3K/mTOR inhibitor dactolisib, prevented leptomeningeal dissemination, via downregulation of UBE2C.

The identification of UBE2C as a molecular marker of prognosis in brain metastatic disease across multiple cancer types and the ability to prevent leptomeningeal dissemination by UBE2C indirect targeting, open a new avenue for the designing of clinical trials toward the prevention of late-stage metastatic brain cancer.

Dissemination of cancer cells to the brain is a frequent and late-stage complication of many systemic cancers. Patients with brain metastatic disease have a poor prognosis, with a median survival between 3 and 11 months.^1,2^ Lung cancer (40%-60%), breast cancer (15%-30%), and melanoma (5%-15%) are the most common primary malignancies that disseminate to the brain.^2–5^ Autopsy studies showed that around 20%-26% of all cancer patients develop brain metastases (BM) but it is thought that the incidence may be higher and increasing.^3,5,6^ Advanced methods of BM diagnosis and more effective treatments for extracranial disease led to an improvement in patients’ survival, thus contributing to an increase in BM incidence. The standard of care treatment for BM includes surgical resection, whole-brain radiotherapy (WBRT), and stereotactic radiosurgery (SRS).^7^ Nonetheless, brain metastatic disease remains incurable.^3^ Furthermore, the most aggressive form of this disease is characterized by coating of the leptomeninges by cancer cells (leptomeningeal dissemination), and poor response to intensive chemotherapeutic regimens, including intrathecal therapy.^8^ Currently, molecular-targeted therapies in use for primary tumors lack good therapeutic responses in metastatic tumors located in the central nervous system (CNS). This might be explained either by the limited crossing of drugs through the blood-brain barrier (BBB)^9^ or by genetic differences between primary tumors and BM.^10^

Understanding the molecular mechanisms underlying the dissemination of cancer cells into the brain will open the possibility of identifying targets to be used in the development of novel therapies. Previous attempts to study the mechanisms of cancer dissemination to the brain focused mainly on analyzing genes differentially expressed between the most common primary tumors (lung and breast cancer) and their BM. The metastatic compartment often exhibits a distinct genetic profile from the primary tumor.^10^ Specific molecular targets have been found to play a role in the metastatic process to the brain, namely HER2, FOXC1, LEF1, and HOXB9 in lung cancer, COX2, HBEGF, and ST6GALNAC5 in breast cancer, and plasminogen activator (PA) inhibitory serpins in both these tumors.^11–13^

We hypothesized that brain metastatic tumors from diverse histological types share common genetic events which promote cancer cell dissemination and colonization of the brain, and these molecular alterations can be used as therapeutic targets. We performed RNA sequencing in a discovery cohort of thirty BM samples from patients diagnosed with various primary tumors and identified UBE2C as being differentially expressed. UBE2C is a ubiquitin-conjugating enzyme that functions together with anaphase-promoting complex/cyclosome (APC/C) involved in the cell cycle, ensuring a correct transition from metaphase to anaphase.^14,15^ UBE2C is present in the 20q13.1 locus, a region known to be amplified in a variety of malignancies, including gastro-esophageal carcinomas.^16^ High expression of UBE2C has been found in tumor tissues from different histological types, and it was associated with a worse prognosis in primary cancers.^16,17^ We analyzed an independent cohort of 89 BM from different primary origins and observed that patients with higher expression of UBE2C had a significantly worse prognosis. In orthotopic mouse models of BM, UBE2C promoted leptomeningeal dissemination and decreased survival, possibly due to an increase in cancer cell migration and invasion. Importantly, early treatment with a PI3K/mTOR inhibitor (dactolisib) prevented the UBE2C-induced leptomeningeal dissemination in these models, revealing a promising therapeutic avenue in advanced metastatic cancer prevention.

## Materials and Methods

### Key Resources

A full description of Materials and methods can be found in Supplementary Materials document.

### Patients’ Samples

BM samples were collected in accordance with the Ethics board from Hospital de Santa Maria (Refª. Nº 367/18 and Refª. Nº 346/20) and a written informed consent was obtained from all patients, prior to study participation.

### RNA Sequencing Analysis

The BM samples from diverse primary origins included in the study reflect the series of patients submitted to brain surgery at the Department of Neurosurgery (HSM-CHULN). Total RNA isolated from BM was processed using the TruSeq RNA Sample Preparation v2 kit (low-throughput protocol; Illumina, San Diego, CA, USA) to prepare the barcoded libraries. Libraries were validated and quantified using either DNA 1000 or high-sensitivity chips on a Bioanalyzer (Agilent, Santa Clara, CA, USA). 7.5 pM denatured libraries were input into cBot (Illumina), followed by deep sequencing using HiSeq 2500 (Illumina) for 101 cycles, with an additional seven cycles for index reading. For normal tissue samples, we downloaded the call sets from the ENCODE portal (https://www.encodeproject.org/) for tissues matching the tissue of origin of the BM.

Fastq files were imported into Partek Flow (Partek Incorporated, St. Louis, MO, USA). Quality analysis and quality control were performed on all reads to assess read quality and to determine the amount of trimming required (both ends: 13 bases 5ʹ and 1 base 3ʹ). Trimmed reads were aligned against the hg38 genome using the STAR v2.4.1d aligner. Unaligned reads were further processed using Bowtie 2 v2.2.5 aligner. Finally, aligned reads were combined before quantifying the expression against the ENSEMBL (release 84) database using the Partek Expectation-Maximization algorithm. Partek Flow default settings were used in all analyses. Files were then processed using Partek Genomic Suite (Partek Incorporated, St. Louis, MO, USA). Genes were filtered for expression values≤1 and 3 or more missing values, the remaining genes were then log2 transformed.

### Microarray Datasets used in the Bioinformatic Analysis

Microarray datasets were also used in the analysis of RNA sequencing. GSE2109 and GSE7307 datasets were downloaded from GEO DataSets (https://www.ncbi.nlm.nih.gov/gds) and processed using Partek Genomic Suite. Files were imported into Partek Genomic Suites and normalized using the RMA method.

### Tissue Microarrays (TMAs)

TMAs were kindly provided by the Neuropathology lab of HSM-CHULN. Protein levels were assessed by immunohistochemical (IHC) staining with UBE2C antibody (Boston Biochem, Cat# A650), ASF1B (Cell Signaling Technology, Cat# 2902), FoxM1 (Cell Signaling Technology, Cat# 5436)or Ki-67 (D2H10) (Cell Signaling Technology, Cat# 9027). For UBE2C, ASF1B and FoxM1 we used semi-quantitative scores of intensity (low or high staining intensity) and frequency (low: 0%-49% staining or high: 50%-100% staining), performed by two independent researchers and validated by a pathologist. For Ki67, an automated software was used to quantify the percentage of positive nuclei (ImmunoRatio).

### Cell Culture

Human cell lines MDA-MB-231 (referred as MDA), A549 and HCT116 were maintained in the appropriate media. We induced brain tropism in these cell lines as previously described.^12^ All cell lines were genetically modified to overexpress UBE2C and MDA, additionally, for the KD of UBE2C.

MET-CF78 cells were derived from a patient with lung cancer BM (patient-derived cell culture), established in our laboratory as previously described.^18^

### Cell Modulation

To achieve stable overexpression, the lentiviral vector was used for gene delivery. The plasmid for human *UBE2C* overexpression was made by subcloning the PCR-amplified UBE2C (IDT) fragment into the EcoRI and Mscl (NEB) site of LeGO-iV2.

The lentiviral vector pLV hU6-sgRNA hUBC-dCas9-KRAB-T2A-Puro (a gift from Charles Gersbach, Addgene plasmid # 71236) was used for stable KD. sgRNAs were obtained from IDT. After annealing, sgRNAs were ligated to BsmBI digested pLV hU6-sgRNA hUBc-dCas9-KRAB-T2a-Puro vector.

Target cells (MDA, A549, and HCT) were stably transduced and selected by flow cytometry using BD FACSAria III cell sorter or 1 µg/ml puromycin (InvivoGen, San Diego, USA, #ant-pr-1).

### Immunoblotting

Whole-cell lysates were prepared as previously described^19^ and the immunoblotting was performed according to standard procedures. Two independent experiments were performed.

### Cell Proliferation

CellTiter 96 Aqueous One Solution Reagent (MTS) was used as defined by the manufacturer’s protocol, for the time points of 0, 24, 48, 72, and 96 hours. Three independent experiments, with 3 technical replicates.

### Colony Formation Assay

MDA or A549 were seeded onto 6 well plates, with 100 cells/well, and incubated at 37°C, 5% CO_2_. Cells were allowed to grow for two weeks. Pictures were analyzed using ColonyArea plugin for ImageJ.^20^

### Migration

Seeding 2×10^4^ cells in CIM-plate 16 and the impedance signals were recorded using the xCELLigence,^21^ for 72 hours.

### Invasion

Seeding of 3×10^4^ cells in matrigel-coated transwells for 30 h, at 37° C and 5%CO_2_.

### In Vivo Orthotopic Xenografts

In accordance with Directive 2010/63/EU (transposed to Portuguese legislation through Decreto-Lei No. 113/2013, of August 7th), all animal procedures were approved by the institutional animal welfare body (ORBEA-iMM). NSG mice were acquired from Charles River Laboratories or in the NSG colony established in-house. Animals were subjected to procedures between the ages of 11 and 22 weeks old. Cancer cells were injected intracranially. MDA models were established by injecting 50,000 cells and 100,000 in MET-CF78 model. Histopathologic analysis was performed in CNS samples.

### Mice In Vivo Imaging

Mice were injected with XenoLight D-Luciferin, Potassium Salt and imaged after 10 minutes in IVIS Lumina System, 5 minutes exposure.

### Histopathological Analysis of Mouse Samples

H&E slides were blindly examined by two independent researchers and a specialized pathologist. Tumor score was defined as 0-no tumor, 1-mild, 2-moderate, 3-marked. Leptomeningeal dissemination score was 0-no neoplasic cell infiltration, 1-mild: minimal focal neoplasic cell infiltration in the meninges, 2-moderate: minimal multifocal neoplasic cell infiltration, 3-marked: high density multifocal neoplasic cell infiltration.

### Drug Screening

A series of six-nine dilution steps of each inhibitor between 32.5 and 25,000 nM was tested. About 30 µl of cell suspension were seeded into each well of the drug library. After 72 hours of incubation at 37°C and 5% CO_2_, plates were analyzed using CellTiter-Glo reagent and read in the Spark MultiMode Plate reader.

### In Vitro Drug Testing

In vitro drug assays were performed by seeding MDA, A549 (1000 cells), or MET-CF78 (500 cells) in 96-well plates and using dactolisib or Genz-644282 (25 nM, 100 nM, 250 nM, 500 nM, and 1 mM). Proliferation assays were performed as described above.

### Drug Testing in Orthotopic BM Xenografts

NSG mice injected intracranially with MDA cancer cell line (5×10^4^ cells/mouse) or MET-CF78 (1×10^5^ cells/mouse) were treated with dual ATP-competitive PI3K and mTOR inhibitor dactolisib (30 mg/kg, based on the literature^22^), via oral gavage, daily from day 4 to day 14, for MDA, or from day 7 to day 18, with a wash-out period of 2 days after 5 days of treatment. Animals were randomized into treatment (dactolisib) and control (vehicle: *N*-Methyl-2-Pyrrolidone (NMP) and Polyethylene glycol 300 (PEG300), 10/90, v/v) groups. By the end of the treatment (day 15 for MDA cells, or 18 for MET-CF78 cells), CNS and organs (lungs, liver, spleen, and kidneys) were collected for histopathological analysis.

### UBE2C IHC in Orthotopic BM Xenografts Samples

In mice injected with MDA and treated with dactolisib (or vehicle), UBE2C downregulation was analyzed by an in-house developed macro, using the ImageJ/Fiji macro to quantify the percentage of high UBE2C-staining in the tumor tissue (available at https://github.com/ClaraBarreto/UBE2C).

### Statistical Analysis

For statistical differences using multiple testing, as in the RNA sequencing analysis, a Bonferroni adjusted *p-*value was used. Other statistical differences were determined using *t*-test (parametric) or Mann–Whitney tests (nonparametric) on GraphPad Prism v6.0 (GraphPad, California, USA, GraphPad Prism, RRID:SCR_002798), as stated in figure legends Differences were considered statistically significant for *p*≤0.05.

## Results

### *UBE2C* is Upregulated in Human BM

**Figure 1.**
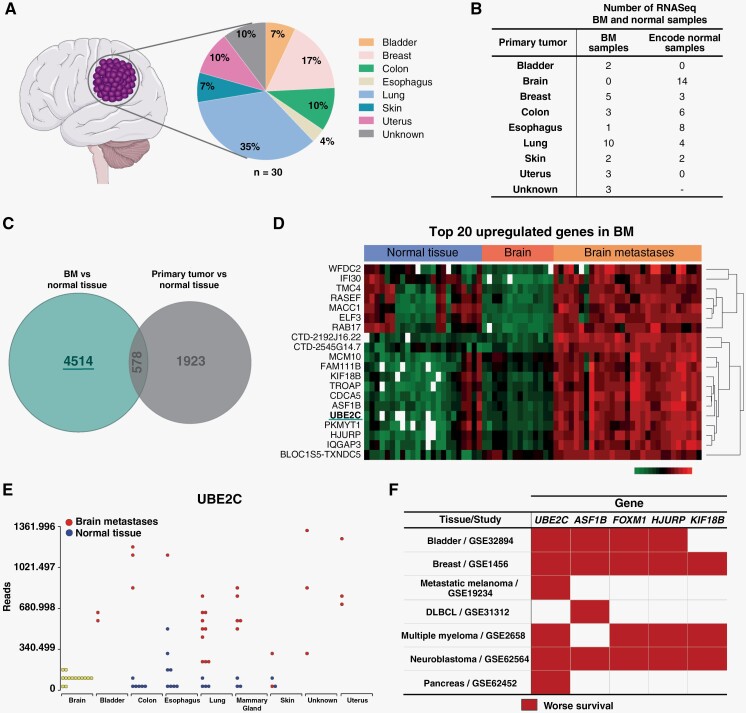
UBE2C is highly expressed in human BM. (A) Percentage of BM included in the RNA sequencing analysis, according to primary tumor origin (*n*=30, discovery cohort from HSM-CHULN). (B) Datasets included in the RNA sequencing (RNASeq) bioinformatic analysis, including data from normal tissue samples, from publicly available datasets. (C) Venn diagram analyzing the differentially expressed genes in BM and primary tumor samples, against genes from normal tissue. (D) Heatmap representing the top 20 upregulated genes in our discovery cohort when compared to publicly available data from normal brain and other normal tissues. (E) Comparison of RNA UBE2C levels in BM and normal tissues. (F) Top 5 upregulated genes in BM and their association with the overall survival (OS) of cancer patients, using datasets from several studies with publicly available RNAseq data: GSE32894: Hoglund, 2015^49^; GSE1456: Bergh, 2005^50^; GSE19234: Bhardwaj,^51^ 2009; GSE31312: International DLBCL Rituximab-CHOP Consortium^52^; GSE2658: Hanamura, 2006^53^; GSE62564: Tong, 2014^54^; GSE62452: Hussain, 2016.^55^ DLBCL: Diffuse large B-cell lymphoma. Red color represents high gene expression significantly associated with decreased OS. The Figure was partly generated using Servier Medical Art, provided by Servier, licensed under a Creative Commons Attribution 3.0 unported license (smart.servier.com).

A discovery cohort from the neurosurgery department at Hospital de Santa Maria from Centro Hospitalar Universitário Lisboa Norte (HSM-CHULN) comprising thirty human BM from patients diagnosed with different primary tumor origins were analyzed using RNA sequencing. In this cohort, lung cancer was the most frequent primary tumor, followed by breast, uterus, and colon cancer (Figure 1A). Tumor samples were collected consecutively, and therefore, the representation of each primary tumor origin reflects the frequency in the neurosurgical series. The transcriptomic analysis included publicly available RNA sequencing datasets of normal tissues (Figure 1B) and microarray datasets of normal tissue and primary tumors (Supplementary Figure S1A), matching the BM cohort histological types. Genes upregulated exclusively in BM samples were identified when compared with normal tissue and primary tumors (*n*=4514 genes, Figure 1C). From this list, we selected the top 20 upregulated genes in BM based on *p*-value≤0.05 and positive or negative fold change≥1.7 (Figure 1D and Supplementary Figure S1B). The expression of these genes was then checked for directionality in all tumor types and the most promising genes were further investigated regarding their clinical relevance. Among the top 5 candidate genes (*UBE2C*, *ASF1B*, *FOXM1*, *HJURP*, and *KIF18B*), *UBE2C* was the most frequently associated with poor prognosis in publicly available clinical datasets from diverse cancer types, including metastatic melanoma (Figure 1E). *UBE2C* was found to be highly expressed in BM samples from diverse primary tumor origins when compared to normal tissues (Figure 1F).

### Patients with High Expression of UBE2C in BM have a Poorer Prognosis

**Figure 2.**
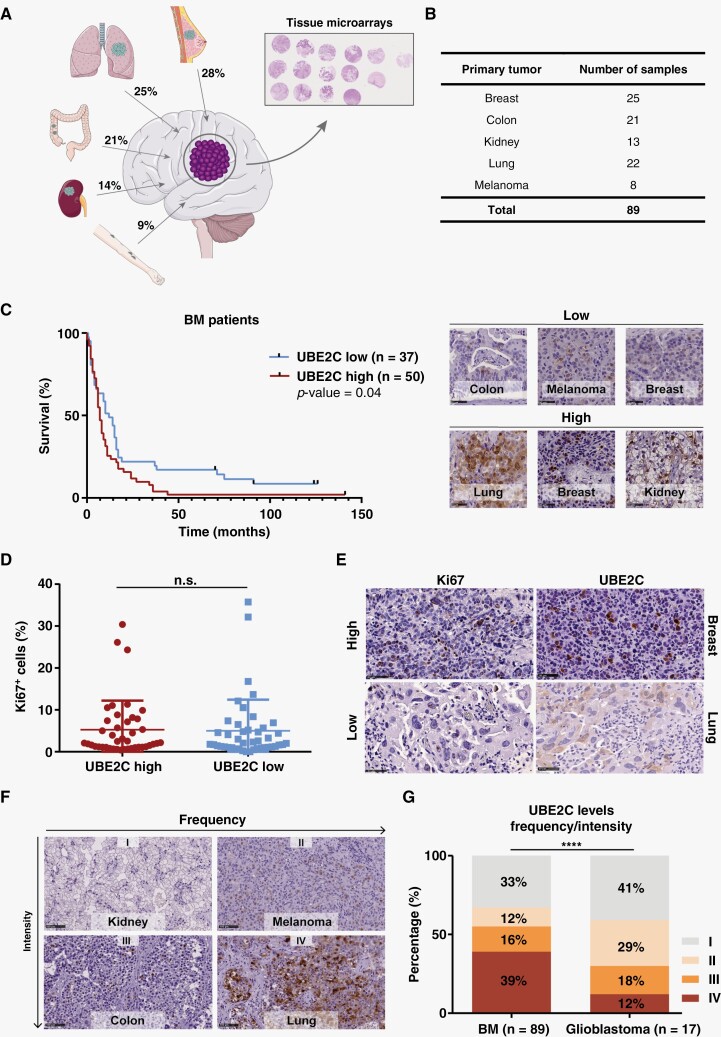
High expression of UBE2C is associated with worse survival in patients with BM. (A) Percentage of BM by tumor type included in the tissue microarrays (TMA). (B) Number of BM samples by tumor origin in the TMA (independent validation cohort, *N*=89). (C) Kaplan-Meier analysis of patients’ survival according to UBE2C protein intensity levels and representative images of the IHC intensity score used (high and low). Differences were considered statistically significant for *p-*value≤0.05, according to the Log-rank (Mantel-Cox) test. (D) Comparison of UBE2C intensity and Ki67 staining in patients with BM. Data is expressed as median with interquartile range. Mann–Whitney test. (E) Representative images of IHC staining for UBE2C (scored as high or low) and Ki67 (assessed using ImageJ); Scale bar: 50 μm. (F) IHC score including intensity and frequency of the UBE2C staining in tumor tissue: I—low/low; II—low/high; III—high/low; III—high/high, respectively; Scale bar: 100 μm. (G) UBE2C expression was compared between BM and brain primary tumors (glioblastoma); ****p-value<0.0001, Chi-square (and Fisher’s exact) test. The Figure was partly generated using Servier Medical Art, provided by Servier, licensed under a Creative Commons Attribution 3.0 unported license (smart.servier.com).

We validated the clinical relevance of UBE2C by immunohistochemistry (IHC) in tissue microarrays (TMAs), using an independent cohort of 89 patients with BM from different primary tumor origins (Figure 2A-B). Samples from patients with primary brain tumors (glioblastoma) were used as control. Patients had a median age of 62 years (28-90 years) (Supplementary Figure S2A), with a similar frequency of male and female patients (52% and 48%, respectively) (Supplementary Figure S2B). The median overall survival of these patients since the diagnosis of BM was 8 months (Supplementary Figure S2C), with different survival rates depending on the primary tumor origin (Supplementary Figure S2D-E). No differences were found in the survival of BM patients when comparing patients with high vs low expression levels (intensity of staining) of ASF1B (Supplementary Figure S2F) or FoxM1(Supplementary Figure S2G). In contrast, high expression of UBE2C (higher intensity of staining) was associated with a worse prognosis (median survival of 7 months), while patients with low UBE2C expression exhibited a median survival of 12 months (*p-*value=0.04) (Figure 2C). This finding was not due to a higher proliferative index in tumor cells (Figure 2D-E), nor dependent on the primary tumor type (Supplementary Figure S2H-L). Using a scoring system combining intensity and frequency of UBE2C staining (Figure 2F), we have observed that most BM samples (55%) have high levels of UBE2C (Score III and IV), in contrast with glioblastomas in which 70% of the samples have low expression of this protein (Figure 2G). This suggests that high UBE2C expression is a feature of brain metastatic tumors, but not of primary brain tumors, such as glioblastoma.

### UBE2C Promotes Cancer Cell Migration and Invasion In Vitro

**Figure 3.**
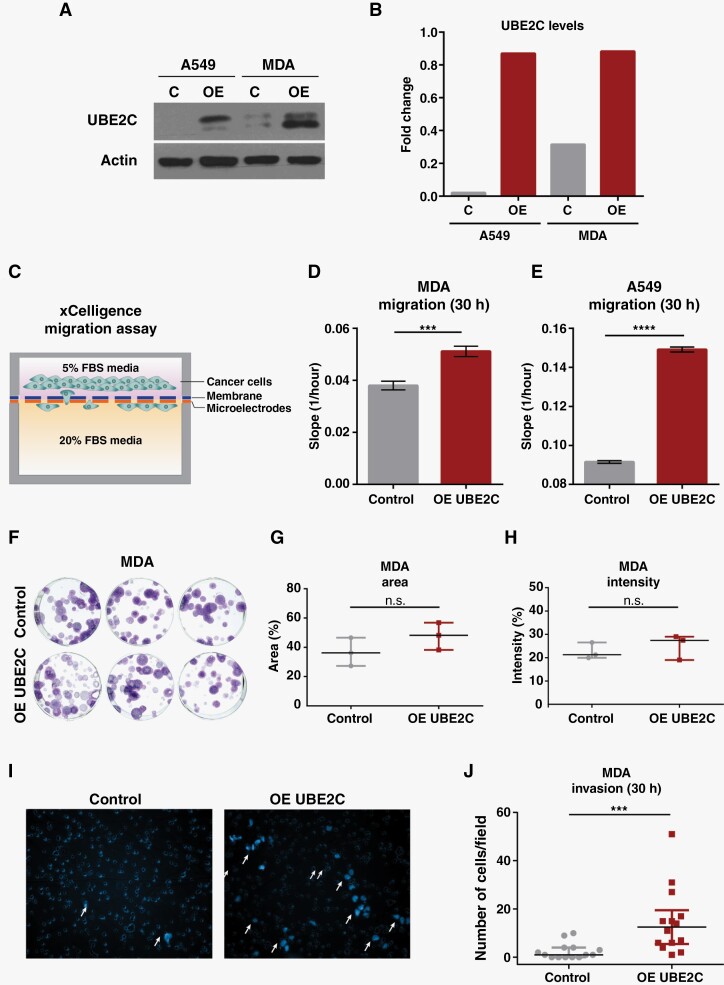
Overexpression of *UBE2C* increases cancer cell migration and invasion. (A) Western Blot of UBE2C levels in MDA and A549 cell lines modulated for overexpression (OE) of *UBE2C* and (B) its densitometry analysis. (C) Schematic representation of migration assay using CIM-Plates in the xCELLigence system. Migration ability of (**D**) MDA and (**E**) A549 cells with OE of *UBE2C* was evaluated using the xCELLigence system at 30h and compared with control cells (empty vector); ****p-value<0.0001, ***p-value=0.001, unpaired t-test. Data represented as mean with SD. (F) Pictures of colony formation assay (CFA) performed in MDA OE *UBE2C* cells (100 cells/well) after 2 weeks, and (G) quantification of the area; *p*-value=0.23 and (H) intensity *p*-value=0.52 of the formed colonies. Mann-Whitney test. Data is presented as median and interquartile range. (I) Representative images and (J) quantification of the invasion capacity of MDA cells with OE *UBE2C* using matrigel-coated transwells at 30 h and compared with control cells (empty vector); ***p-value=0.0002, Mann-Whitney test. Data are presented as median with interquartile range.

To validate and characterize the role of UBE2C in the context of brain metastatic disease we used two cancer cell lines, MDA (breast cancer) and A549 (lung cancer). Both cell lines were engineered to stably overexpress *UBE2C* (Figure 3A-B). To study the effect of UBE2C in cancer cell migration, we performed real-time cell analyses using the xCELLigence system (Figure 3C). The migration rate of MDA and A549 cells was significantly increased in UBE2C overexpressing cells (Figure 3D-E). This effect was not due to an increase in proliferation either in short-term (Supplementary Figure S3A-B) or long-term assays (Figure 3F-H and Supplementary Figure S3C-E), as assessed by MTS and colony formation assays, respectively. In addition, UBE2C increased the invasion ability of MDA cells in matrigel-coated transwells (Figure 3I-J).

### UBE2C Drives Leptomeningeal Dissemination and Associates with Worse Survival In Vivo

**Figure 4.**
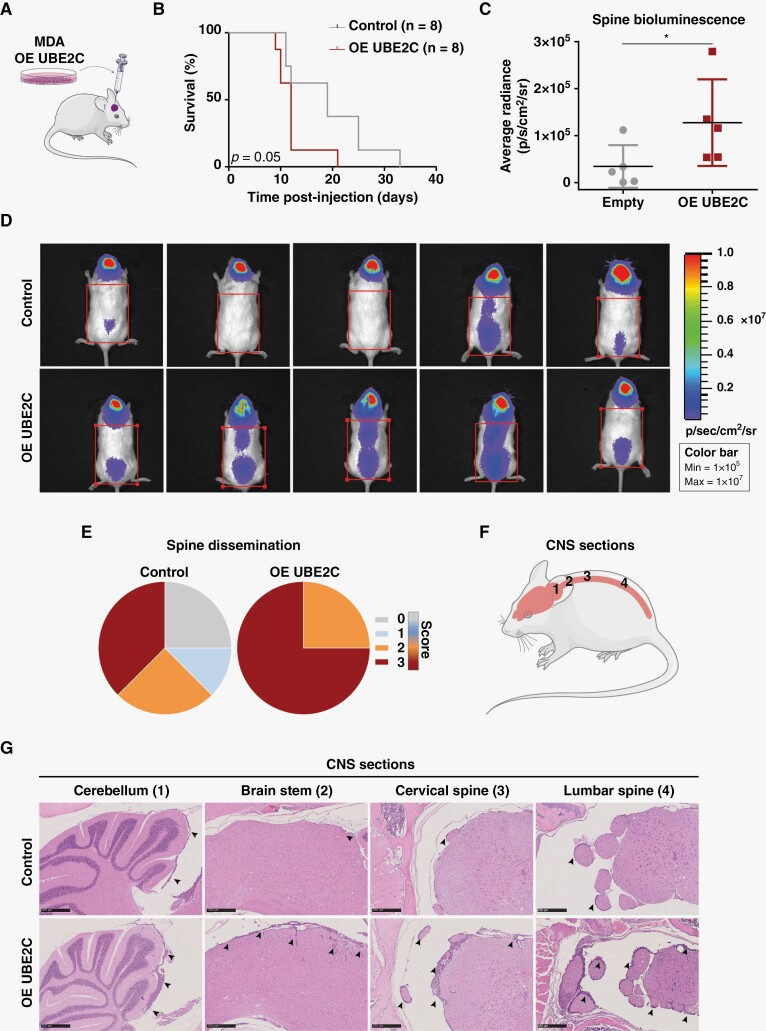
*UBE2C* decreases survival and mediates leptomeningeal dissemination driving an aggressive disease phenotype in vivo. (A) Intracranial injection of NSG mice (*n*=8/group) with MDA cell line, control and OE *UBE2C;* (B) and the survival analysis of both groups, *p-value=0.05, Kaplan-Meier test. (C) Representative images ofthewhole-body bioluminescence imaging of control and OE *UBE2C* mice at day 9 post-injection, and (D) quantification of the average radiance signal in the spine; *p-value=0.03, Mann-Whitney test. Data represented as median with interquartile range. (E) Spine leptomeningeal dissemination in animals injected with control and OE *UBE2C* cells. Histopathological score used to assess the leptomeningeal dissemination: 0- negative; 1- mild; 2- moderate; 3- marked. (F) CNS sections analyzed by histopathology, and (G) representative images of these sections with H&E staining (5x; scale bar: 250 μm) from mice injected with control and OE *UBE2C* cells. Black arrows indicate leptomeningeal dissemination. The Figure was partly generated using Servier Medical Art, provided by Servier, licensed under a Creative Commons Attribution 3.0 unported license (smart.servier.com).

To assess the in vivo role of UBE2C in BM, we performed intracranial injections of MDA cells in NSG mice (Figure 4A). Animals injected with *UBE2C*-overexpressing cells showed decreased survival (Figure 4B, *p*-value=0.05), a phenotype associated with more aggressive disease, characterized by leptomeningeal dissemination. Although the levels of leptomeningeal dissemination in the brain were similarly high in both experimental groups (Supplementary Figure S4A), we observed a significant increase in spinal cord dissemination among animals with *UBE2C*-overexpressing cells, both in vivo (Figure 4C-D) and ex vivo (Figure 4E-G). We also established an MDA cell line with *UBE2C* knockdown (KD) using the CRISPR system (Supplementary Figure S4B). There were no differences in survival (Supplementary Figure S4C) or dissemination to the brain meninges (Supplementary Figure S4D) in orthotopic models transplanted with *UBE2C* KD or control cells. However, we found evidence of decreased dissemination of cancer cells with *UBE2C* silencing to the spinal cord meninges (Supplementary Figure S4E). We also observed that lung cancer sublines with tropism to the leptomeninges have increased levels of UBE2C in comparison with their parental counterparts (Supplementary Figure S4F-G). Thus, *UBE2C* drives a more aggressive disease phenotype with leptomeningeal dissemination and decreased survival in orthotopic models of BM.

### Targeting the PI3K/mTOR Pathway In Vitro Decreases Cancer Cell Proliferation and Downregulates UBE2C

**Figure 5.**
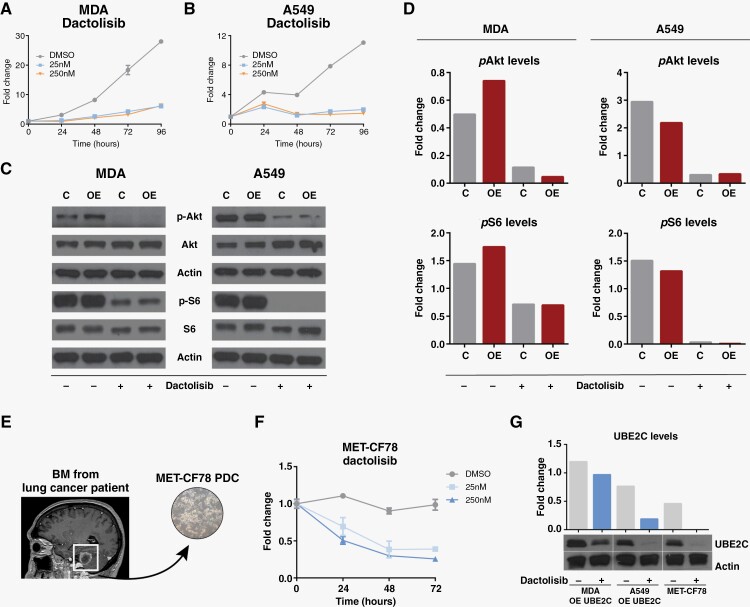
Downregulation of PI3K/mTOR pathway inhibits cancer cell proliferation and decreases UBE2C levels. MTS assays using 25 nM and 250nM concentrations of dactolisib to assess proliferation in (**A**) MDA and (**B**) A459 cell lines with OE of *UBE2C*. (C) Evaluation of the effect of dactolisib in PI3K/mTOR pathway (*p*-Akt and *p*-S6 levels) by western blot using MDA and A549 cells with *UBE2C* overexpression (OE) in comparison with control cells (D). (E) MET-CF78 PDC derived from a BM of a lung cancer patient. (F) Inhibition of proliferation by dactolisib in patient-derived cultures from a lung cancer BM (MET-CF78). (G) UBE2C levels assessed by western blot in cancer cell lines treated with dactolisib (250 nM) for 24 h and its densitometry analysis.

Due to the lack of effective treatment options for patients with BM and the inexistence of specific therapies targeting UBE2C, we performed a high-throughput drug screening to identify compounds with efficacy in treating UBE2C-driven BM. We used a chemical library of 650 compounds, FDA approved or in phase 3 or 4 clinical trials, to test cell lines with overexpression and knock-down of UBE2C (Supplementary Figure S5A). In this drug screening, we have selected two candidate compounds that targeted cells expressing high levels of UBE2C when compared to their counterparts, for further in vitro validation: dactolisib (PI3K/mTOR inhibitor) and Genz644282 (Topoisomerase I inhibitor) (Supplementary Figure S5B-C). Both compounds proved to be highly effective inhibiting cancer cell proliferation (Figure 5A-B and S5D-E).

Single-cell RNA sequencing data analysis of BM from patients with different primary tumors^23^ showed that metastatic tumor cells with high *UBE2C* expression levels (Supplementary Figure S5F) also presented high levels of *MTOR1* (Supplementary Figure S5G), with a positive correlation between the mRNA levels of both genes (Supplementary Figure S5H). In addition, the PI3K/mTOR pathway has been implicated in brain metastatic cancer.^10^ Therefore, we decided to further evaluate the efficacy of inhibition of PI3K/mTOR signaling and examine its interplay with UBE2C. Dactolisib effectively inhibited the PI3K/mTOR pathway in breast and lung cancer cell lines in vitro, through the downregulation of pAkt and pS6, respectively (Figure 5C-D). Furthermore, we have also used a patient-derived culture (PDC), MET-CF78, isolated from a BM-derived from a lung cancer patient^18^ with constitutive expression of UBE2C (Figure 5E). We observed inhibition of MET-CF78 proliferation by dactolisib in a dose-dependent manner (Figure 5F). Interestingly, the targeting of PI3K/mTOR signaling pathway by dactolisib decreased the UBE2C levels in all cancer cell lines (Figure 5G), regardless of their different levels of PI3K activation (Supplementary Figure S5I).

### Dactolisib Prevents Leptomeningeal Dissemination In Vivo

**Figure 6.**
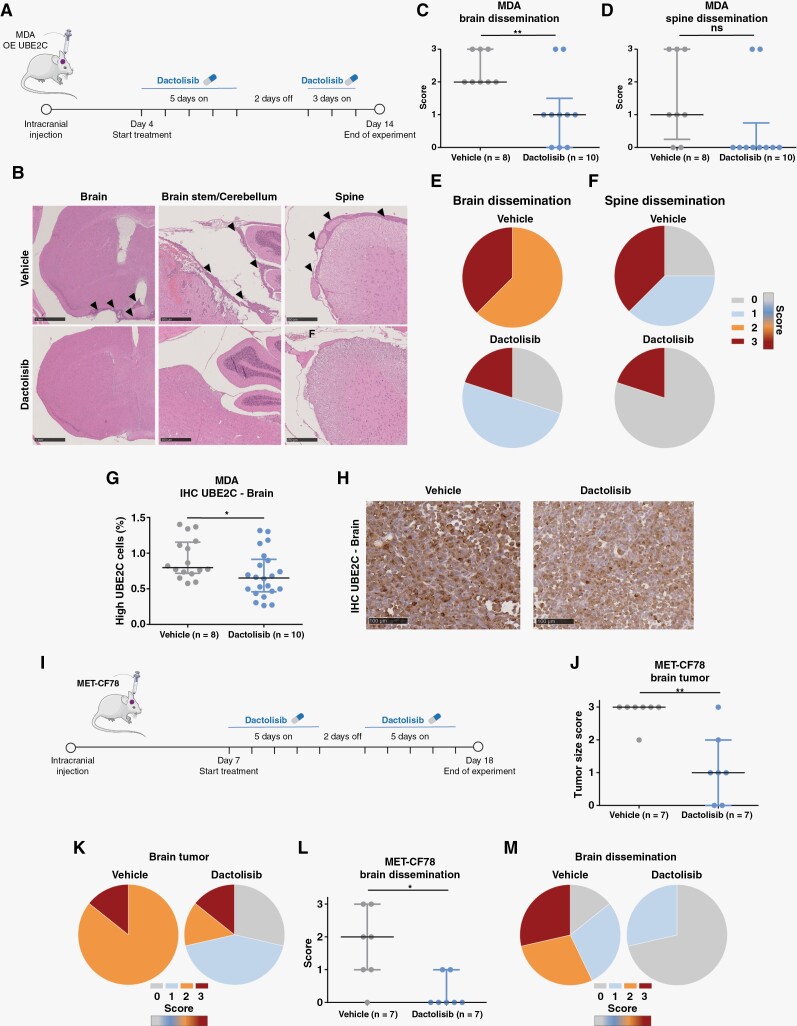
PI3K/mTOR dual inhibitor dactolisib prevents leptomeningeal dissemination in *UBE2C*-drivenmouse models of BM. (A) Treatment protocol in orthotopic xenografts using breast cancer cells (MDA) with overexpression of *UBE2C*. Animals were treated daily from day 4 to day 14 post-injection with dactolisib (30 mg/kg; n=10) or vehicle (NMP and PEG300, 10%-90%, v-v; *n*=8), with a wash-out period of 2 days. (B) Representative images of leptomeningeal dissemination in the control group and absence of dissemination in the dactolisib-treated group. Brain (scale: 1 mm), brain stem/cerebellum (scale: 500 μm), and spine (scale: 250 μm). (C) Histopathological scoring of leptomeningeal dissemination in the brain (**p-value=0.0083, t-test, data represented as mean and SD) and (D) in the spine (*p*-value=0.07, Mann-Whitney test, data represented as median and interquartile range). (E) Percentage of brain dissemination in vehicle (2: 62,5%, 3: 37,5%) and treatment (0: 30%, 1: 50%, 3: 20%) groups. (F) Percentage of spine dissemination in vehicle (0: 25%, 1: 37,5%, 3: 37,5%) and treatment (0: 80%, 3: 20%) groups. (G) Expression of UBE2C by IHC staining, in brain tumor samples upon treatment with dactolisib. *p-value=0.03, *t*-test. Data represented as mean with SD. (H) Representative images of UBE2C staining in brain tumor sections. (I) Treatment protocol in patient-derived xenografts of lung cancer BM (MET-CF78) (*n*=7/group). Animals were treated daily from day 7 to day 17 post-injection with dactolisib or vehicle, with a wash-out period of 2 days; Scale bar: 100 μm. (J) Histopathological scoring of the brain tumor size (0: no tumor; 1: minimal to mild; 2: moderate; 3: marked); **p-value=0.009; Mann-Whitney test. Data represented as median with interquartile range, and (K) brain tumor size score in vehicle (2: 14,286%, 3: 85,714%) and dactolisib (0: 28,571%, 1: 42,857%, 2: 14,286%, 3: 14,286%) treated mice. (L) Histopathological scoring of brain dissemination as described above; *p-value=0.026, Mann-Whitney test; data represented as median with interquartile range, and (M) percentage of brain dissemination in vehicle (0: 14,286%, 1: 28,571%, 2: 28,571%, 3: 28,571%) and treatment (0: 81,429%, 1: 28,571%) groups. Score used to assess the leptomeningeal dissemination: 0- negative; 1- mild; 2- moderate; 3- marked. The Figure was partly generated using Servier Medical Art, provided by Servier, licensed under a Creative Commons Attribution 3.0 unported license (smart.servier.com).

We then asked whether the aggressive metastatic phenotype induced by UBE2C in vivo could be prevented by early treatment with dactolisib, a compound known to cross the BBB.^24^ A preclinical therapeutic protocol was designed to early treat UBE2C-driven orthotopic mouse models of BM with dactolisib by oral gavage. Treatment was initiated on day 4 post-injection, and it was given daily for 10 days, with a wash-out period of two days (Figure 6A). There were no differences in weight between dactolisib-treated and untreated animals (Supplementary Figure S6A). Strikingly, although dactolisib was not able to significantly reduce brain tumor size (Supplementary Figure S6B), it was effective in preventing leptomeningeal dissemination both in the brain and spine (Figure 6C-F), reversing the aggressive phenotype induced by *UBE2C*-overexpressing cancer cells. Dactolisib-treated brain tumors exhibited a significant decrease in the number of cancer cells expressing high levels of UBE2C (Figure 6G-H).

In the patient-derived xenograft of the lung cancer BM (MET-CF78), early preclinical treatment with oral dactolisib (Figure 6I) had no differences in spine dissemination (Supplementary Figure S6C) and there was no impact of the treatment in the weight of the animals (Supplementary Figure S6D). However, dactolisib significantly reduced both brain tumor size (Figure 6J-K) and leptomeningeal dissemination to the brain (Figure 6L-M).

To validate if the effect of dactolisib in vivo was, at least in part, *UBE2C*-dependent, we developed orthotopic mouse models using MDA cells with KD of *UBE2C* (*n*=7 mice/group). There were no differences in the weight of treated animals compared to the untreated (Supplementary Figure S6E). In UBE2C-depleted tumors, dactolisib did not significantly impact brain tumor size (Supplementary Figure S6F) or CNS dissemination (Supplementary Figure S6G-H).

## Discussion

Despite significant therapeutic advances in the treatment of primary cancers, BM remain a major clinical hurdle. We began addressing this challenge by interrogating BM from diverse primary cancers using RNA sequencing coupled with functional approaches, in both human and mouse models. Focusing on BM from diverse primary tumors, we were able to identify common genetic events leading to cancer cell dissemination and/or colonization into the brain. Simultaneously, these molecular targets constitute novel targets for therapy.

We identified UBE2C as a differentially expressed gene in human BM and showed that high levels of UBE2C are associated with shorter survival in cancer patients with brain metastatic disease. UBE2C was first described by Okamoto as an oncogene, overexpressed in primary tumors and cancer cell lines, when compared to normal tissues.^17^ It has also been shown that UBE2C correlates with higher tumor grades. In lung cancer patients, UBE2C was associated with poorer survival^25^ and with tumor progression, as a consequence of autophagy inhibition and increased tumor invasiveness.^26^ In breast cancer, UBE2C was identified as a prognostic marker^27^ and associated with cancer grade progression^28^ and drug resistance.^29^ Similar observations were made in colon, ovarian, and bladder cancers, among other types of tumors.^30–33^ Interestingly, UBE2C was associated with BM in a recent publication, where single-cell RNA sequencing of BM from multiple cancers led to the identification of two subgroups, being UBE2C one of the signature genes in the proliferative group.^23^

UBE2C has been described as a promoter of migration and invasion of tumor cells in endometrial cancer models, inducing epithelial to mesenchymal transition (EMT), via downregulation of p53 levels.^34^ Similar effects were also observed in gastric^35^ and hepatocellular cancers.^36^ We observed that orthotopic mouse models injected with cancer cells overexpressing UBE2C developed a more aggressive disease phenotype, with leptomeningeal dissemination in the brain and spinal cord. This phenomenon may be explained by the increase in cancer cell migration and invasion induced by overexpression of this gene, as observed in vitro. One possible explanation for the increased colonization of the leptomeninges, particularly in the spinal cord, in a poor microenvironment such as the cerebrospinal fluid (CSF), is that cancer cells with high levels of UBE2C have higher glycolytic activity, further promoting their migration and invasion abilities even in such environmental conditions.^37,38^ In fact, a metabolic diagnostic tool based on nuclear magnetic resonance (NMR) analysis of patients with leptomeningeal carcinomatosis showed increased lactate levels, a product of glycolysis.^39^

In cancer patients, leptomeningeal dissemination can occur in approximately 5%-15% of the cases,^8^ having a dismal prognosis with a median survival between 2 and 4 months.^8,40–42^ Very little is known about the molecular biology of this advanced stage of metastatic cancer, making it exceedingly difficult to treat. Aggressive therapies including intrathecal chemotherapy and whole-brain radiation have failed to alter the natural history of leptomeningeal dissemination. Recent phase 2 studies have reported the clinical benefit of patients treated with EGRF inhibitors and immunotherapy,^43,44^ although the duration of responses in leptomeningeal disease was limited in time.

Since late-stage CNS dissemination seems very difficult to target, the best chance for improved survival in these patients would be to prevent cancer cells from disseminating into the leptomeninges. Our observations revealed that early treatment with oral dactolisib (a dual PI3K/mTOR inhibitor) prevented the development of leptomeningeal dissemination in orthotopic mouse models driven by UBE2C. This effect of dactolisib might be UBE2C-dependent because, in UBE2C-depleted tumors, treatment did not reduce brain tumor size or CNS dissemination.

Moreover, dactolisib effectively reduced dissemination and brain tumor size in a patient-derived xenograft from a lung cancer BM. Dactolisib brain bioavailability and clinical effect have been previously reported, even at lower doses.^45,46^ In both in vivo models, the dual PI3K/mTOR inhibitor was well tolerated and did not induce toxicity. Interestingly, mutations in PI3K/Akt/mTOR signaling have been found in BM, but not in primary tumors.^10^ Also, activation of this signaling pathway has already been associated with leptomeningeal dissemination in melanoma patients.^47^ We have shown that PI3K/mTOR pathway inhibition with dactolisib decreased cancer migration and invasion, and downregulated UBE2C both in vitro and in vivo. The connection between PI3K/Akt/mTOR pathway and UBE2C has been previously reported in gastric cancer cells, where UBE2C led to the activation of AURKA and consequent EMT by the decrease in p-AKT1 levels.^35^ Furthermore, the mTOR inhibitor CCI-779 decreased the levels of UBE2C in castration-resistant prostate cancer, by disrupting its transcription and blocking UBE2C-dependent invasion.^48^ We believe dactolisib targets key steps in the metastatic cascade (mobilization of cancer cells from the primary tumor and invasion) and, therefore, may be considered as a novel therapeutic strategy to prevent brain metastatic disease.

Further studies are warranted to better explore the mechanisms of UBE2C-driven dissemination to the brain and to dissect the cellular interplay between cancer, stroma, and immune cells, mediated by UBE2C. Moreover, the limited size of our BM patients’ cohort and the number of models used for validation (breast and lung cancer) do not allow the generalization of our results for each primary cancer type. The comparison of UBE2C expression levels in BM with matched primary tumors or extracranial metastases would also add layers of information on UBE2C specificity. Additional functional studies should be performed to validate if the leptomeningeal dissemination driven by high levels of UBE2C is a general feature of cancers from different primary origins, using other BM cancer models, besides breast and lung cancer.

This notwithstanding, we have shown that UBE2C is a relevant player in brain metastatic disease, especially relevant for leptomeningeal dissemination, and that it can be used as a prognostic marker in cancer patients with this condition. Furthermore, we demonstrated that UBE2C-induced leptomeningeal dissemination can be prevented in vivo by targeting the PI3K/mTOR pathway. The results from our study may prompt the advancement of PI3K/mTOR inhibitors into clinical trials for patients with advanced metastatic cancer of the CNS from multiple primary origins.

## Supplementary Material

vdad048_suppl_Supplementary_Figure_S1

vdad048_suppl_Supplementary_Figure_S2

vdad048_suppl_Supplementary_Figure_S3

vdad048_suppl_Supplementary_Figure_S4

vdad048_suppl_Supplementary_Figure_S5

vdad048_suppl_Supplementary_Figure_S6

vdad048_suppl_Supplementary_Figures

vdad048_suppl_Supplementary_Material
