# Supplementary figures and images for: UBE2C promotes leptomeningeal dissemination and is a therapeutic target in brain metastatic disease
Source: Neurooncol Adv. 2023 Apr 28;5(1):vdad048. doi: 10.1093/noajnl/vdad048 (PMC10195208; doi:10.1093/noajnl/vdad048)

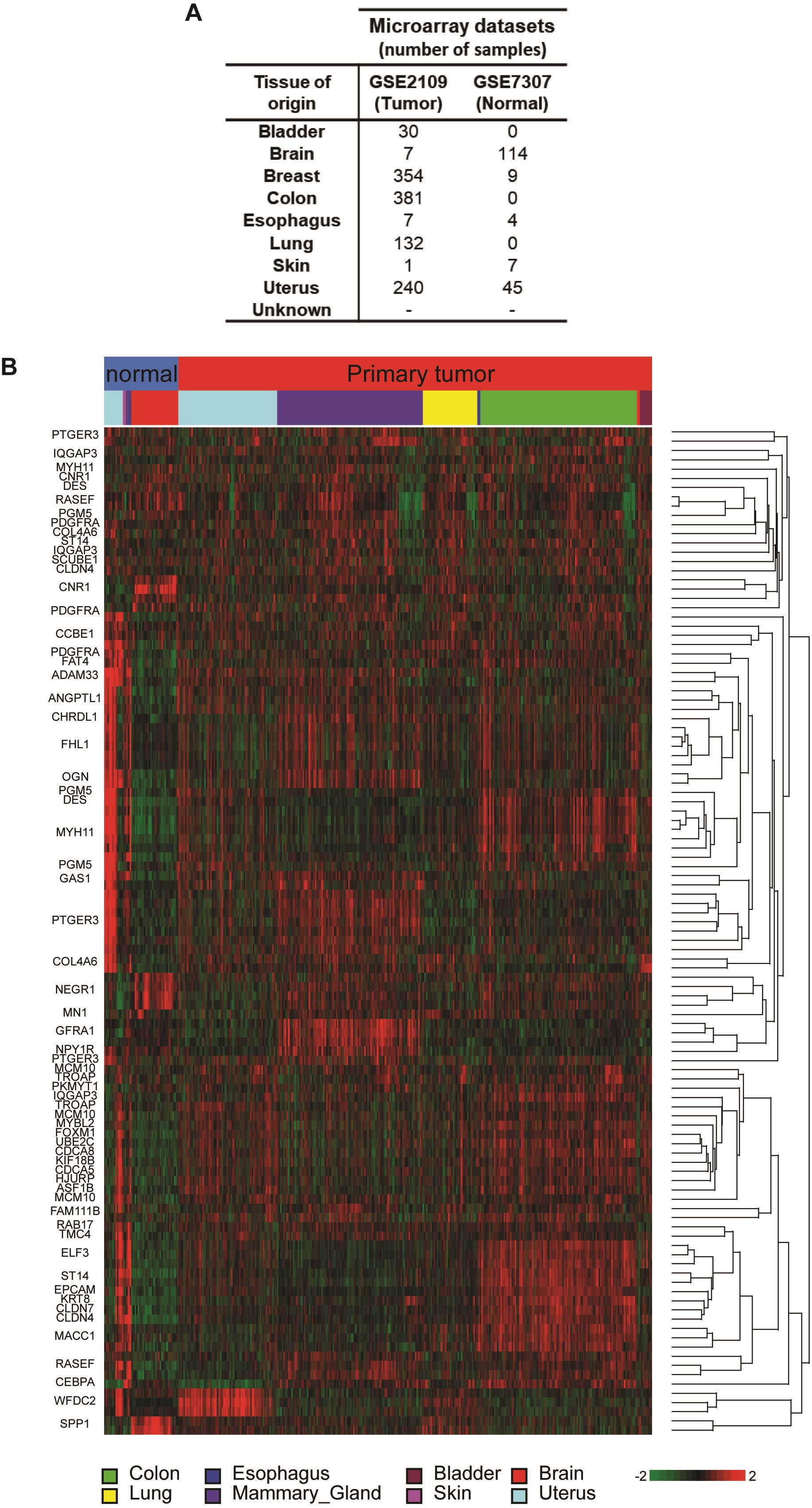

Supplement: vdad048_suppl_Supplementary_Figure_S1 [file vdad048_suppl_supplementary_figure_s1.jpeg]

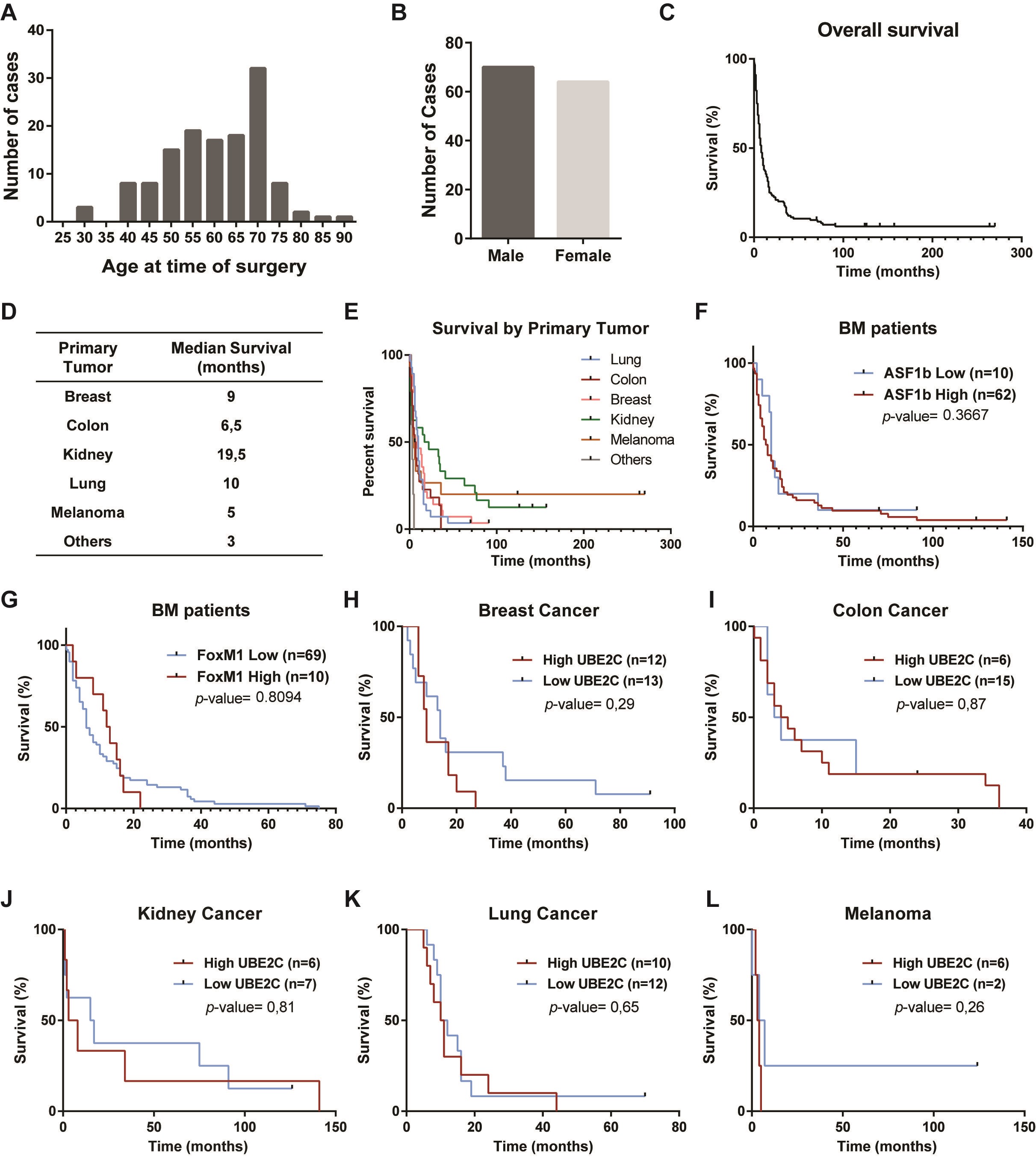

Supplement: vdad048_suppl_Supplementary_Figure_S2 [file vdad048_suppl_supplementary_figure_s2.jpeg]

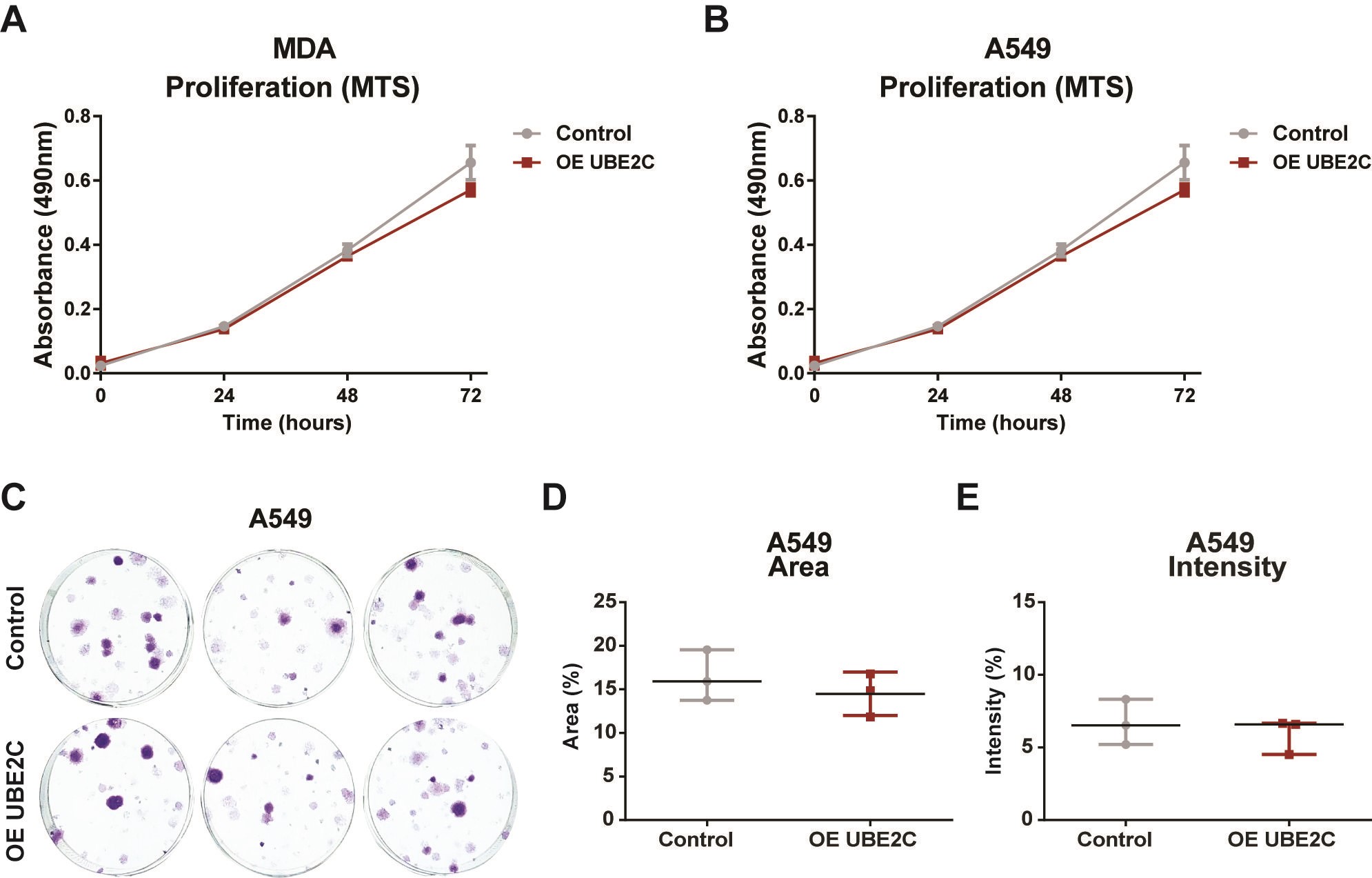

Supplement: vdad048_suppl_Supplementary_Figure_S3 [file vdad048_suppl_supplementary_figure_s3.jpeg]

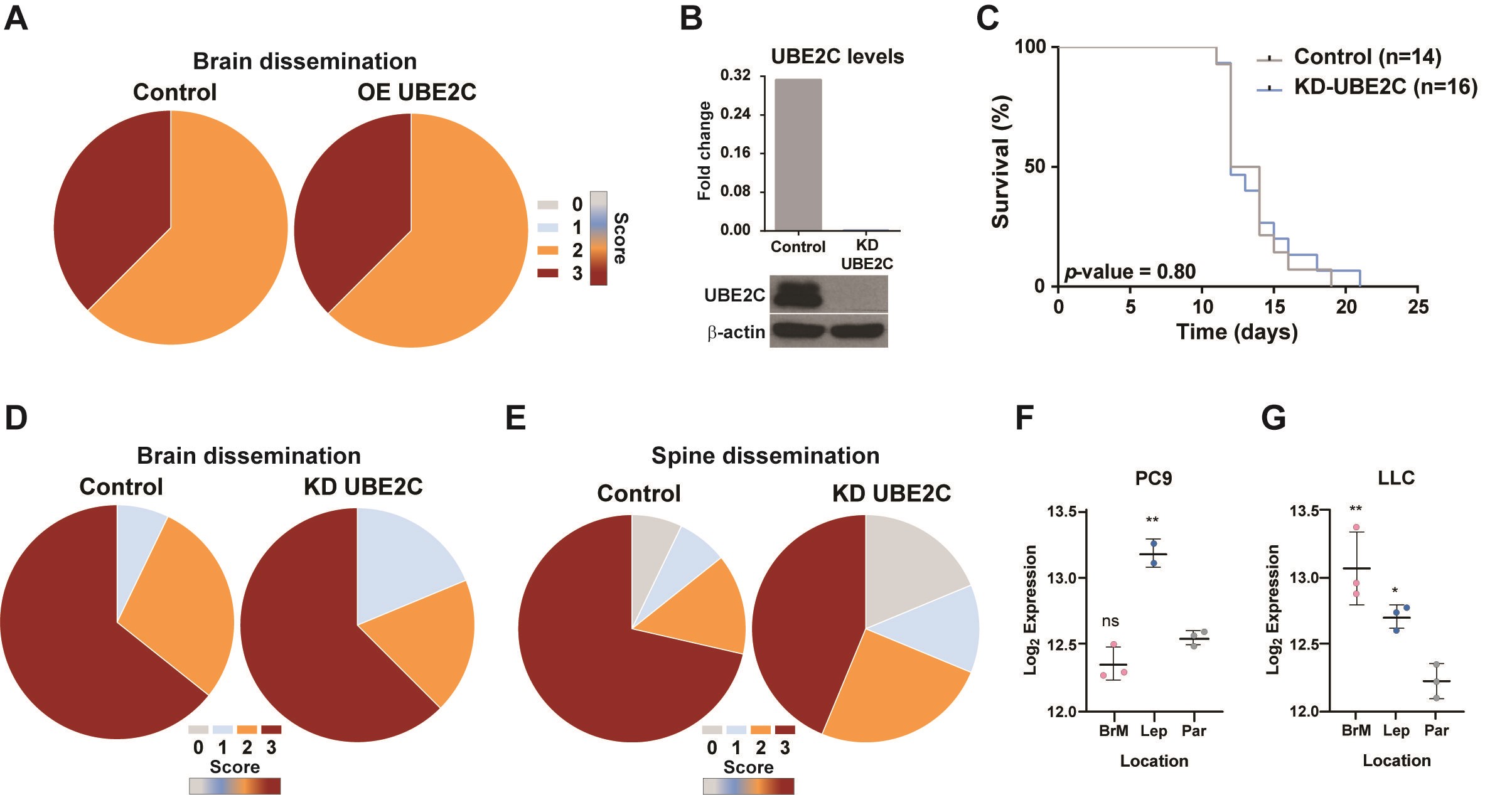

Supplement: vdad048_suppl_Supplementary_Figure_S4 [file vdad048_suppl_supplementary_figure_s4.jpeg]

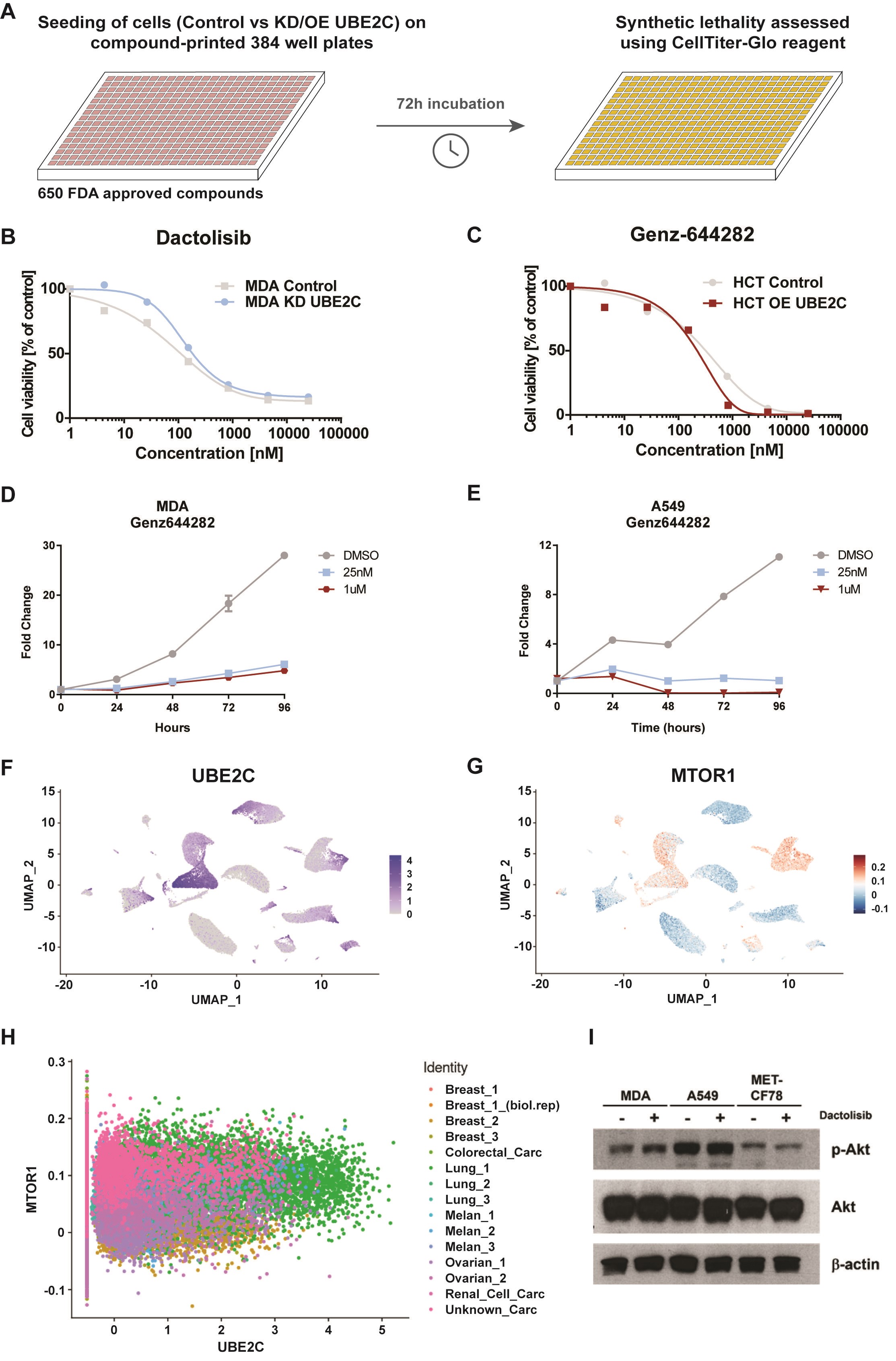

Supplement: vdad048_suppl_Supplementary_Figure_S5 [file vdad048_suppl_supplementary_figure_s5.jpeg]

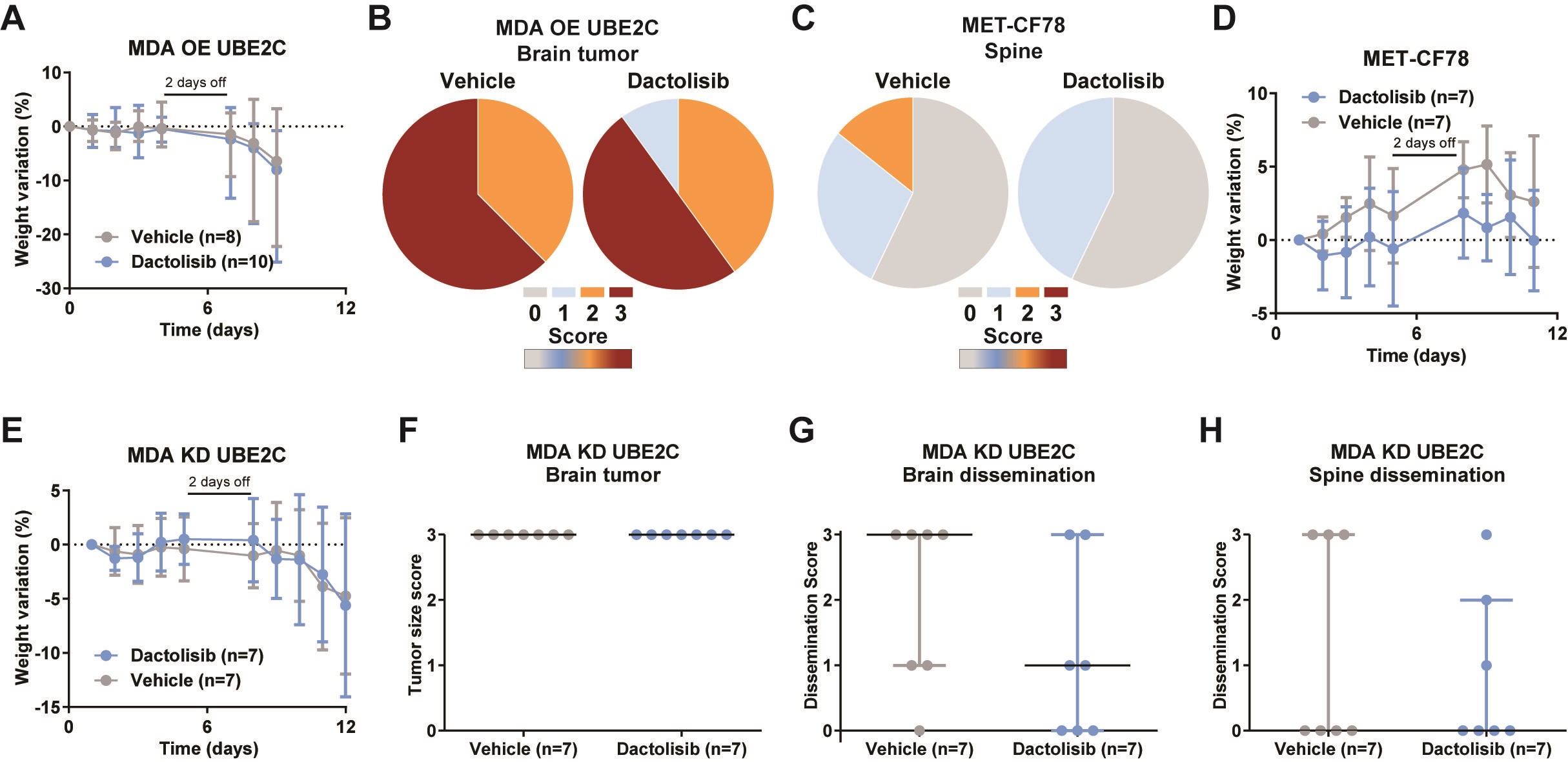

Supplement: vdad048_suppl_Supplementary_Figure_S6 [file vdad048_suppl_supplementary_figure_s6.jpeg]
