# Supplementary material for: UBE2C promotes leptomeningeal dissemination and is a therapeutic target in brain metastatic disease
Source: Neurooncol Adv. 2023 Apr 28;5(1):vdad048. doi: 10.1093/noajnl/vdad048 (PMC10195208; doi:10.1093/noajnl/vdad048)
Supplement: vdad048_suppl_Supplementary_Material [file vdad048_suppl_supplementary_material.docx]

**SUPPLEMENTARY MATERIALS AND METHODS**

## **Patients’ samples**

BM samples and detailed clinical data were collected from 30 patients with diverse primary tumors (lung, breast, uterus, bladder, colon, esophagus and melanoma), submitted to surgical resection in the Department of Neurosurgery at HSM-CHULN. Surgical specimens not needed for diagnostic purposes were snap frozen in liquid nitrogen and stored at Biobanco-iMM CAML (biobank of Lisbon Academic Medical Center, Lisbon, Portugal) within an hour after surgery. BM samples were collected in accordance with the Ethics board from Hospital de Santa Maria (Refª. Nº 367/18 and Refª. Nº 346/20) and a written informed consent was obtained from all patients, prior to study participation. Samples were then requested from Biobanco-iMM for RNA sequencing analysis, as described below.

## **RNA isolation from BM specimens**

Snap frozen samples of BM were used in the RNA sequencing analysis. Total RNA was isolated from BM tissue (500ng) using Trizol^TM^ reagent (Invitrogen-Thermo Fisher Scientific, Waltham, Massachusetts, USA, Cat#15596026) according to the manufacturer’s recommendations. Following isolation, RNA was stored in RNase/DNase-free water at −80°C. The quantity and quality of the isolated RNA was assessed by NanoDrop^TM^ (Thermo Fisher Scientific, Waltham, Massachusetts, USA). Samples were excluded if the yield did not reach the minimum requirement of 1000ng or RIN>6 (RNA Integrity Number).

## **RNA sequencing analysis**

Total RNA isolated from BM was processed using the TruSeq RNA Sample Preparation v2 kit (low-throughput protocol; Illumina, San Diego, CA, USA) to prepare the barcoded libraries. Libraries were validated and quantified using either DNA 1000 or high-sensitivity chips on a Bioanalyzer (Agilent, Santa Clara, CA, USA). 7.5 pM denatured libraries were input into cBot (Illumina), followed by deep sequencing using HiSeq 2500 (Illumina) for 101 cycles, with an additional seven cycles for index reading. For normal tissue samples, we downloaded the call sets from the ENCODE portal (https://www.encodeproject.org/) for tissues matching the tissue of origin of the BM.

Fastq files were imported into Partek Flow (Partek Incorporated, St. Louis, MO, USA). Quality analysis and quality control were performed on all reads to assess read quality and to determine the amount of trimming required (both ends: 13 bases 5’ and 1 base 3’). Trimmed reads were aligned against the hg38 genome using the STAR v2.4.1d aligner. Unaligned reads were further processed using Bowtie 2 v2.2.5 aligner. Finally, aligned reads were combined before quantifying the expression against the ENSEMBL (release 84) database using the Partek Expectation-Maximization algorithm. Partek Flow default settings were used in all analyses. Files were then processed using Partek Genomic Suite (Partek Incorporated, St. Louis, MO, USA). Genes were filtered for expression values ≤1 and 3 or more missing values, remaining genes were then log2 transformed.

## **Microarray datasets used in the bioinformatic analysis**

Microarray datasets were also used in the analysis of RNA sequencing. GSE2109 and GSE7307 datasets were downloaded from GEO DataSets (https://www.ncbi.nlm.nih.gov/gds) and processed using Partek Genomic Suite. Files were imported into Partek Genomic Suites and normalized using the RMA method.

## **Tissue Microarrays (TMAs)**

Tissue microarrays (TMAs) were previously built in the Neuropathology lab of HSM-CHULN and kindly provided by Pedro Pereira and Professor José Pimentel. The TMA included samples of BM collected from patients with primary tumors from diverse origins (**Fig. 2A**). Protein levels were assessed by immunohistochemical (IHC) staining with UBE2C antibody (Boston Biochem, Cat# A650), ASF1B (Cell Signaling Technology, Cat# 2902), FoxM1 (Cell Signaling Technology, Cat# 5436)or Ki-67 (D2H10) (Cell Signaling Technology, Cat# 9027), using 5μm sections of the formalin fixed, paraffin embedded (FFPE) TMA. FFPE sections were incubated with the primary antibody and with EnVision+ (Dako, Glostrup, Denmark). Color was developed in solution containing diaminobenzadine-tetrahydrochloride (Sigma, Missouri, USA), 0.5% H_2_O_2_ in phosphate-buffered saline buffer (pH 7.6). Slides were counterstained with hematoxylin and mounted. IHC evaluation was performed blindly by 2 independent researchers and a specialized pathologist. For UBE2C we used a semi-quantitative score of intensity (low or high staining intensity) and frequency (low: 0-49% staining or high: 50-100% staining). For Ki67, an automated software was used to quantify the percentage of positive nuclei (ImmunoRatio). Images were acquired using a NanoZoomer SQ slide scanner (Hamamatsu Photonics, Hamamatsu City, Japan) with 20x magnification (0.46μm resolution).

## **Cell culture cell lines and induction of brain tropism *in vivo***

Human cell lines MDA-MB-231 (referred as MDA), A549 and HCT116 were maintained in the appropriate media and passaged up to 15 times. MDA-MB-231 were cultured in DMEM 1x (Gibco-Thermo Fisher Scientific, Waltham, Massachusetts, USA, Cat# 41966-029), supplemented with FBS 10% (BioWest, Cat# S1810-500) and L-glutamine (Gibco, Cat #25030-024). Same media was used for A549, further supplemented with non-essential amino acids (Gibco, Cat# 11140-035). HCT116 were cultured in McCoy’s (Gibco, Cat# 26600-023), supplemented with 10% FBS. We have induced brain tropism in these cell lines as previously described^12^. Briefly, GFP/Luc positive cells were injected intracardially in NSG mice and collected once brain metastases were established. After sorting the dissociated cells, these were cultured and re-injected intracardially in mice to generate cells lines with brain tropism (Br). We generated Br derivatives of MDA-MB-231, A549 and HCT116, described from now on as MDA, A549 and HCT, respectively. All cell lines were genetically modified to overexpress UBE2C and MDA for the KD of UBE2C.

MET-CF78 cells were derived from a patient with lung cancer BM (patient-derived culture, PDC) established in our laboratory^18^, as previously described. Briefly, cancer cells were isolated by enzymatic dissociation of a tumor derived from a subcutaneous PDX model which was implanted with BM sample from a patient with lung cancer. MET-CF78 were cultured in DMEM-F12 media (Gibco #11320-074) supplemented with 2% B-27, 1% N2 supplement, 1% L-glutamine (Gibco, Cat# 25030-024), 1x antibiotic-antimycotic (Gibco, Cat # 15240-096), rh-FGF (Stem Cell Technologies, Cat #02634) and rh-EGF (Sigma, Cat #E9644). Cells were seeded in previously coated flask with poly-L-ornithine and laminin.

## **Cell modulation**

To achieve the stable overexpression, the lentiviral vector was used for gene delivery. LeGO-iV2 (a gift from Boris Fehse, Addgene plasmid #27344) (Weber et al., 2008) was used to construct the recombinant lentiviral vector. The plasmid for human *UBE2C* overexpression was made by subcloning the PCR-amplified UBE2C (IDT) fragment into the EcoRI and Mscl (NEB) site of LeGO-iV2.

The lentiviral vector pLV hU6-sgRNA hUBC-dCas9-KRAB-T2A-Puro (a gift from Charles Gersbach, Addgene plasmid # 71236) was used for stable KD. sgRNAs were obtained from IDT. After annealing, sgRNAs were ligated to BsmBI digested pLV hU6-sgRNA hUBc-dCas9-KRAB-T2a-Puro vector.

For lentiviral transfection, HEK293T cell lines were seeded in Petri dishes. Twenty-four hours after seeding, cells were incubated with lentiviral vector, helper, envelope plasmids, and polyethylenimine (PEI, Merck, Darmstadt, Germany, Cat# 408727) to increase the efficiency of infection. Cell culture medium containing the virus was collected 24 and 48 hours after infection. Target cells (MDA, A549 and HCT) were then infected with the produced virus using 2µg/ml polybrene (Merck, Darmstadt, Germany, #TR-1003-G). Stably transduced cells were either selected by flow cytometry using BD FACSAria III cell sorter or 1µg/ml puromycin (InvivoGen, San Diego, USA, #ant‐pr‐1).

## **Immunoblotting**

Whole cell lysates were prepared as previously described^19^. Briefly, cells were lysed in lysis buffer supplemented with 1x phosphatase inhibitors (PhosStop, Roche Diagnostics, Basel, Switzerland) and a 1x protease inhibitor cocktail (Complete Mini, Roche Diagnostics, Basel, Switzerland). After centrifugation at 10000g for 15 minutes at 4ºC, the supernatant was harvested. Total protein concentration was determined using the Bradford protein assay (BioRad, California, USA). Equal amounts of protein were subjected to sodium dodecyl sulfate–polyacrylamide gel electrophoresis and transferred onto nitrocellulose membranes (BioRad, California, USA), which were blocked with 5% skim milk for 1 hour at room temperature, incubated with specific primary antibodies overnight at 4ºC. Immunodetection was performed by incubation with horseradish-peroxidase–conjugated appropriate secondary antibodies and developed by chemiluminescence (Curix60, AGFA). The antibodies used were as follows: UBE2C (Boston Biochem, Cat# A650) and beta-actin (Abcam, Cat# ab8224). The latter was used as loading control. Two independent experiments were performed. The densitometry analysis was performed using Adobe Photoshop® software.

## **Cell proliferation**

Cell proliferation rate was determined at 24h, 48h, 72h and 96h using the CellTiter 96 Aqueous One Solution Reagent (MTS) (Promega, Wisconsin, USA, Cat# G3581) as defined by the manufacturer’s protocol. Briefly, in each time-point cells were plated in 96 well plates and incubated at 37ºC for 2h with MTS. The absorbance was measured at 490nm using the microplate reader Infinite M200 (Tecan, Crailsheim, Germany). Three independent experiments were performed with 3 technical replicates each.

## **Colony Formation Assay**

MDA or A549 single cell suspensions were seeded onto 6 well plates, with 100 cells per well, and incubated at 37ºC, 5% CO2. Cells were allowed to grow for two weeks in order to form single-cell-derived colonies. Wells were washed three times with PBS and fixed in 0.4% formaldehyde overnight and stained with 0.5% gentian violet for 2 minutes at room temperature. Staining was washed in distilled water, submerging the plates, and then allowed to air dry. Pictures of the plates were analyzed using ColonyArea plugin for ImageJ^20^ which calculates area and intensity of staining in colony formation assays.

## **Migration**

Cancer cells were seeded on the top chamber (2x10^4^ cells) of a CIM-Plate 16 (OMNI Life Science GmbH & Co KG, Bremen, Germany; Cat# 2801038) in triplicates. Cell migration was assessed in the xCELLigence system (ACEA Biosciences)^21^ in real time for 45 hours and readings were recorded every 15 minutes. Cells maintained in serum-free media served as a control.

## **Invasion**

Invasion assays were performed using 6.5mm Transwell® with 8.0µm pore size polyester membrane insert (Corning, New York, USA, Cat# 3464), coated with Matrigel® Matrix (Corning, New York, USA, Cat# 356237). Thirty thousand cancer cells were seeded on the top chamber in media with 5% FBS and the bottom chamber had 20% FBS media. Cells were allowed to migrate for 30h, at 37ºC and 5%CO_2_. After incubation, both chambers were carefully washed with PBS-Tween (0.05%), fixed with methanol (2 minutes at -20ºC) and stained with DAPI (Thermo Fisher Scientific, Waltham, Massachusetts, USA Cat# D1306) for 5 minutes at room temperature. Five random fields of each condition were acquired using Zeiss Axio Observer equipment (Zeiss, Jena, Germany), and the number of cells was counted manually in the Zen Blue software.

## ***In vivo* orthotopic xenografts**

In accordance with Directive 2010/63/EU (transposed to Portuguese legislation through Decreto-Lei No. 113/2013, of August 7th), all animal procedures were approved by the institutional animal welfare body (ORBEA-iMM), in order to ensure that the use of animals complies with all applicable legislation and following the 3R's principle, as well as licensed by the Portuguese competent authority (license number: 012028\2016). All animals were kept in specific pathogen-free (SPF) conditions, randomly housed per groups under standard laboratory conditions (at 20-22°C under 10hour light/14hour dark), and given free access to food (RM3, SDS Diets, Witham, UK) and water (Ultrapure). Invasive procedures were performed with animals under anesthesia (ketamine, Ketamidor 100mg/ml 10ml, Plurivet; Medetomidina, Domtor 1mg/ml 10ml, Ecuphar), administered via intraperitoneal injection. Humane endpoints were established for 10% body weight loss, body condition scores ≤2 or lethargy, ataxia, bleeding, hunched/stiffed posture, self-mutilation, and skin bruising in consequence of tumor burden. Animals were euthanized using anesthetic overdose, using pentobarbital.

NSG mice were purchased from Charles River Laboratories (Massachusetts, USA) or obtained from a NSG colony established in-house. Animals were subjected to procedures between the ages of 11 and 22 weeks old.

Cancer cells were injected intracranially in the frontal region of the cerebral cortex, using the bregma as reference point (2mm lateral right, 0mm anterior, and 2.5mm ventral). MDA cells were established by injecting 50000 cells, while 100000 MET-CF78 cells were injected in this model.

All mice were monitored for body weight, discomfort and distress every other day. Mice were euthanized, once any of the humane endpoints was reached. Histopathologic analysis was performed in CNS samples.

## ***In vivo* imaging of mice**

Mice were imaged under anesthesia. Prior to the image acquisition, animals were injected by intraperitoneal injection with XenoLight D-Luciferin, Potassium Salt (Perkin Elmer, Boston, USA, Cat# PELS122799) subcutaneously. After 10 minutes, bioluminescence images were acquired using IVIS Lumina System using 5 minutes exposure (Perkin Elmer), and analyzed using Living Image software, version 3.0.

## **Histopathological analysis of mouse samples**

Tissue samples were fixed immediately in 10% neutral buffered formalin solution, dehydrated, and embedded in paraffin, serially sectioned at a thickness of 5μm using a microtome, mounted on microscope slides and stained with hematoxylin and eosin (H&E) for morphological examination. H&E slides were blindly examined by two independent researchers and a specialized pathologist, and representative photomicrographs were taken using the NanoZoomer SQ slide scanner (Hamamatsu Photonics, Hamamatsu City, Japan) with 20x magnification (0.46μm resolution).

## **Drug screening**

Drug screening was conducted using Corning 384-well microtiter plates pre-dispensed with inhibitors dissolved in DMSO prior to cell seeding using D300e Drug Dispenser (Tecan, Crailsheim, Germany), sealed using Parafilm and stored at -80°C until use. Stock solutions for printing were prepared at 10mM concentration. A series of six-nine dilution steps of each inhibitor between 32.5 and 25000nM was printed in logarithmic distribution and DMSO content was normalized to 0.25% in all wells. Staurosporine was added to each plate as a positive control. DMSO and empty wells served as negative controls. In addition, to avoid plate effects, the two outer columns and rows were not used, and the inner part was dispensed in a randomized fashion. One hour before testing, the assay plates were removed from ‐80°C and thawed at room temperature.

To improve screening stability, all (MDA and HCT) cell lines underwent a cell density optimization procedure. Using MultiDrop Combi Reagent Dispenser (Thermo Fisher Scientific, Schwerte, Germany), 30µl of cell suspension were seeded into each well of the pre-printed plates. After 72 hours of incubation at 37ºC and 5% CO_2_, plates were taken out of the incubator and equilibrated to room temperature for 30 minutes. Thirty microliters of CellTiter-Glo reagent (Promega GmbH - Walldorf, Germany Cat# G7573) was added to each well and plates were incubated 10 minutes at 37ºC and posteriorly read in the Spark MultiMode Plate reader (Tecan, Crailsheim, Germany).

## ***In vitro* drug testing**

*In vitro* drug assays were performed by seeding MDA, A549 (1000 cells) or MET-CF78 (500 cells) in 96-well plates and using different concentrations (25nM, 100nM, 250nM, 500nM and 1mM) of dactolisib (Selleckchem, Munich, Germany, Cat# S1009) or Genz-644282 (Selleckchem, Munich, Germany, Cat# S0093). Proliferation assays were performed as described above.

## ***In vivo* drug testing using orthotopic BM xenografts**

*In vivo* drug response evaluation was performed using NSG mice injected intracranially with MDA cancer cell line (5x10^4^ cells/mouse) or MET-CF78 (1x10^5^ cells/mouse). Dual ATP-competitive PI3K and mTOR inhibitor Dactolisib (30mg/kg, based on literature^22^) was purchased from Selleck Chemicals (Munich, Germany). Dactolisib was freshly solved in N-Methyl-2-Pyrrolidone (NMP; Sigma-Aldrich, Darmstadt, Germany, Cat# 328634) and Polyethylene glycol 300 (PEG300; Sigma-Aldrich, Darmstadt, Germany, Cat# 202371) (10/90, v/v) immediately before administration by oral gavage, weight-adjusted. Animals were randomized into treatment group and one untreated control group (vehicle: N-Methyl-2-Pyrrolidone (NMP) and Polyethylene glycol 300 (PEG300). Treatment of MDA or MET-CF78-injected mice started four- or seven-days post-injection, respectively, and mice received two cycles of therapy (five days on and two days off), until day 13 or 17 post-injection (**Fig. 6A** and **I**). The animals were monitored daily, and body weight variations were recorded throughout treatment. Mice were euthanized by the end of the treatment (day 15 or 18), and CNS and organs (lungs, liver, spleen and kidneys) were collected for histopathological analysis, as described above.

## **UBE2C IHC in orthotopic BM xenografts samples**

In mice injected with MDA and treated with dactolisib (or vehicle), UBE2C downregulation was analyzed by an in-house developed macro for ImageJ/Fiji. Images of the intracranial tumors were acquired using the Nanozoomer SQ system (acquisition: 20x; export: 40x magnification) and analyzed using the ImageJ/Fiji macro to quantify the percentage of high UBE2C-staining in the tumor tissue (available at https://github.com/ClaraBarreto/UBE2C). Briefly, the total tumorous tissue area (TTA) is determined by using the “Color Threshold” feature in the HSB color space, after manual exclusion of non-tumorous tissue and staining artifacts regions. Two classes of positive staining were then defined: dark brown for strong staining and light brown for weak staining. Threshold values for the “Color Threshold” feature were then empirically determined for the dark-brown area (DBA) using the RGB color space, by analyzing a set of representative images with strong staining. The light-brown area (LBA) was then calculated using the formula: LBA = TTA – DBA, and area fractions were determined as follows: %LBA = LBA/TTA and %DBA = DBA/TTA.

## **Statistical analysis**

For statistical differences using multiple testing, as in the RNA sequencing anlaysis, a Bonferroni adjusted *p-*value was used. Other statistical differences were determined using t-test (parametric) or Mann–Whitney tests (non-parametric) on GraphPad Prism v6.0 (GraphPad, California, USA, GraphPad Prism, RRID:SCR_002798), as stated in figure legends Differences were considered statistically significant for p≤0.05.
